# Multistate Mumps Outbreak Originating from Asymptomatic Transmission at a Nebraska Wedding — Six States, August–October 2019

**DOI:** 10.15585/mmwr.mm6922a2

**Published:** 2020-06-05

**Authors:** Matthew Donahue, Blake Hendrickson, Derek Julian, Nicholas Hill, Julie Rother, Samir Koirala, Joshua L. Clayton, Thomas Safranek, Bryan Buss

**Affiliations:** ^1^Epidemic Intelligence Service, CDC; ^2^Nebraska Department of Health and Human Services; ^3^University of Nebraska Medical Center College of Public Health, Omaha, Nebraska; ^4^South Dakota Department of Health; ^5^Northeast Nebraska Public Health Department, Wayne, Nebraska; ^6^University of Nebraska-Lincoln; ^7^Division of State and Local Readiness, Center for Preparedness and Response, CDC.

In August 2019, 30 attendees at a Nebraska wedding developed mumps after being exposed to one asymptomatic index patient who was fully vaccinated according to Advisory Committee on Immunization Practices (ACIP) recommendations (*1*), resulting in a multistate outbreak. A public health investigation and response revealed epidemiologic links that extended from the index patient through secondary, tertiary, and quaternary patients and culminated in a measles-mumps-rubella (MMR) booster vaccination campaign in the local community where approximately half of the patients resided.

## Investigation and Results

On August 26, 2019, the Nebraska Department of Health and Human Services (NDHHS) was notified by a South Dakota hospital of three suspected mumps cases (awaiting laboratory confirmation) in patients who had attended a wedding in Nebraska on August 3. On August 28, an attendee list including 176 families (approximately 325 attendees) was obtained from the bride. She identified 25 wedding attendees that she believed to be ill, including an attendee who developed symptoms <24 hours after the wedding and 15 days before symptom onset in the next earliest ill person identified. Attendees on the list resided in 14 states: Arizona, Arkansas, Colorado, Georgia, Idaho, Iowa, Kansas, Minnesota, Nebraska, North Dakota, Oklahoma, Pennsylvania, South Dakota, and Wyoming. That same day, NDHHS issued an alert and call for cases using Epi-X to public health partners nationwide that emphasized the potential for the outbreak to reach to multiple states. The following day, statewide Health Alert Network advisories were sent to providers in Nebraska and South Dakota, and a media statement was released in Nebraska.

To identify additional cases, NDHHS developed a web-based questionnaire using Research Electronic Data Capture,* and the link was provided to all 176 attending families by e-mail and letters to ascertain illness status, symptom onset date among ill persons, and symptoms. In addition, reports of potential mumps cases were solicited from health care providers, local health departments, the South Dakota Department of Health, and clinical, commercial, and public health laboratories. Mumps case status was assigned as probable or confirmed using the 2012 Council of State and Territorial Epidemiologists case definition (*2*). Patients, including those identified through the questionnaire, were interviewed by telephone and advised to observe standard mumps isolation precautions (*3*). Self-reported MMR vaccination history was collected from patients during the investigation, and persons with unknown vaccination histories were cross-referenced with state vaccination registries. CDC’s Vaccine Preventable Diseases Reference Center at the Minnesota Public Health Laboratory genotyped four isolates collected from Nebraska patients.

The index patient, a Nebraska resident aged 25 years who worked as a child caretaker, had close contact over a 6-day period beginning July 25 with an ill child aged 1 year who had recently returned from a family vacation in Florida and Antigua.^†^ The child had received the first on-schedule dose of MMR vaccine in June and on return from vacation on July 24, developed a high fever, and exhibited frequent ear-pulling. The child received medical attention on July 24, 26, and 27 and was given a diagnosis of a viral illness. The index patient attended the wedding on August 3 (day 9 after her initial exposure to the child) and reported extensive social interactions, including sharing drinks and dancing. She developed left ear and jaw tenderness the next day (August 4) and parotitis on August 5 (11 days after exposure); she sought medical care on August 9 (day 15). She received treatment with corticosteroids,^§^ but because no diagnostic testing was performed, she was classified as having a probable case of mumps.

The index patient verified that neither the child nor the child’s family attended the wedding and reported she had no contact with any wedding attendees in the weeks preceding the wedding. This index patient was fully vaccinated according to ACIP guidelines (*1*), which was verified in the state vaccination registry. Drinking wine from a shared vessel, a potential vehicle for transmission of respiratory illnesses at weddings, was not a part of the wedding ceremony.

Among approximately 325 persons who attended the wedding, 148 (46%) completed the online questionnaire. Overall, 31 secondary cases (including 13 confirmed and 18 probable) were identified (Figure). Patients with secondary cases reported parotitis onset from August 19 to September 1 (16–29 days after the wedding). Thirty of these patients attended the wedding (attack rate = 30 of 325 [minimum = 9%]); one patient did not attend the wedding but was exposed to the index patient elsewhere. Fourteen patients (45%) resided in community A, a town in northeastern Nebraska with a population of approximately 1,400 persons. Among the 30 patients who attended the wedding, 15 (50%) had received 2 doses of MMR vaccine. Three patients (two who had received 2 doses of MMR vaccine and one with an unknown vaccination history) who had no likely exposures except the wedding developed parotitis 26–29 days after the wedding, which is longer than the typical mumps incubation period of 12–25 days (*3*).

**FIGURE:**
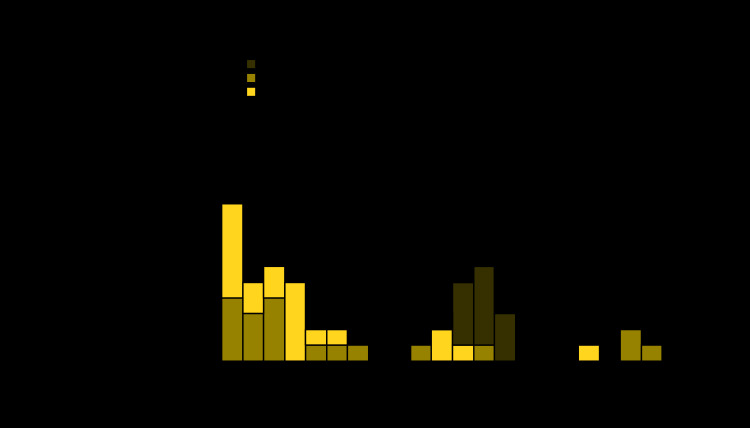
Onset of parotitis among persons with confirmed and probable mumps cases (N = 62) — six states,* August–October 2019 * Community A events included street dance (N = 7), football game (N = 6), school (N = 3), and unspecified (N = 2).

Twenty-seven tertiary cases (23 confirmed and four probable) were subsequently identified. Patients’ reported parotitis onset dates ranged from September 7 to September 23 (35–51 days after the wedding). Seventeen (63%) patients resided in community A. Six cases were epidemiologically linked to secondary cases. Eighteen were linked to different events in community A. Three were community A residents with no other known epidemiologic links.

Three quaternary cases, all confirmed, were identified. Patients’ reported parotitis onset dates ranged from September 26 to September 29 (54–57 days after the wedding). All three resided in community A and were epidemiologically linked to a tertiary case.

In total, 62 cases were identified (39 confirmed and 23 probable); 54 (87%) were Nebraska residents, including 34 (55%) from community A and eight (13%) from other states (three secondary cases among wedding attendees from South Dakota, one tertiary case from South Dakota, and four secondary cases, one each from Idaho, Minnesota, North Dakota, and Wyoming). Median patient age was 35 years (range = 6–59 years, old enough to have received 2 doses of MMR vaccine); 41 (66%) had received ≥2 doses of MMR vaccine (Table), and 37 (60%) were male. No serious mumps complications or hospitalizations were identified. Genotype testing identified isolates of one secondary patient as indeterminate, one tertiary patient as genotype G, and two quaternary patients as genotype G. No additional cases linked to this outbreak have been identified in Nebraska or elsewhere.

**TABLE:** Measles-mumps-rubella (MMR) vaccination histories of mumps patients* (N = 62) — six states, August–October 2019

No. of MMR doses received	No. (%) of patients
0	2 (3)
1	5 (8)
2	38 (61)
3	3 (5)
≥1	51 (82)
Unknown	9 (15)

*Mumps vaccination histories were first self-reported during investigations, and vaccine registries were then queried for persons with unknown vaccination status.

## Public Health Response

With 45% of secondary cases occurring among community A residents, state and local public health officials considered an MMR booster vaccine campaign in that community. Because predicting ongoing transmission was difficult given the point-source nature of the wedding exposure and wide geographic distribution of ill attendees, a communitywide vaccination campaign was not initiated at that time. However, after 63% of tertiary cases were identified among community A residents, the increased perception of ongoing risk for the community and potential benefit of a communitywide MMR booster vaccine campaign warranted an escalated response (*4*).

To inform Community A residents of the vaccination campaign, a flyer was distributed to the Chamber of Commerce, local schools, city and county offices, local radio and television stations, a local cable access television channel, and through the local health department’s Facebook page and websites. The target population was estimated at 700 persons using the American Community Survey (*5*). Thirty public health officials and volunteers participated in the vaccination campaign, and the National Incident Management System for clinic operations was used to structure the event. Residents were screened to determine whether they met criteria to receive the MMR vaccine, including adults aged 19–62 years living or working in community A with no medical contraindications and who had not received a mumps diagnosis or a mumps-containing vaccine within the past 6 months. On October 3, a total of 327 (47%) persons from the target population received an MMR vaccine dose at the community’s fire station.

## Discussion

A mumps outbreak involving six states occurred following exposure to an asymptomatic, fully vaccinated (*1*) index patient who reported extensive social interaction during the peak period of infectivity, in an environment where potentially susceptible persons were densely clustered. Mumps immunity after childhood vaccination can wane by early adulthood (*6*). It is likely that waning of vaccine-induced immunity contributed to this outbreak, because approximately two thirds of patients had received ≥2 doses of MMR vaccine, and the median patient age was 35 years. Specific viral factors (e.g., mutations increasing pathogenicity and shedding) were not a likely contributor because mumps genotype G is commonly implicated in both sporadic cases and outbreaks in the United States (*7*). However, mumps is most infectious just before and during onset of parotitis (*3*), and the timing of the event likely contributed to transmission among exposed attendees because the index patient developed parotitis the day after the wedding. The wedding served as a setting conducive to droplet transmission, facilitated by close social contact.

Isolation of ill persons and a communitywide MMR vaccination campaign helped end the outbreak. As of December 1, 2019, no additional cases had been identified in community A, nor had any additional cases been identified in any other state as linked to this outbreak. A decline in case count before the campaign was observed, which complicated assessment of the campaign’s relative contribution in controlling the outbreak. Collaborative efforts, including early and regular communication between local, state, and national public health authorities, local health care providers, and community officials proved crucial for efficient resource mobilization, strengthened preparedness, and resulted in effective disease containment.

**Summary**

**What is already known about this topic?**

Since 2006, most U.S. mumps cases have been reported among persons who have received 2 doses of measles-mumps-rubella (MMR) vaccine. Mumps is most infectious just before and during the onset of parotitis.

**What is added by this report?**

A multistate outbreak followed contact with an asymptomatic, fully vaccinated index patient who reported extensive social interactions at a wedding, resulting in 31 secondary cases, 27 tertiary cases, and three quaternary cases. Isolation and a communitywide third-dose MMR vaccination campaign helped end the outbreak.

**What are the implications for public health practice?**

Asymptomatic transmission of mumps in a conducive environment is capable of producing a widespread outbreak. An MMR vaccine campaign can be considered in community settings.

## Footnotes

* https://www.project-redcap.org/.

† Antigua is one of the Leeward Islands in the Caribbean region and the main island of the country of Antigua and Barbuda.

§ https://www.cdc.gov/mumps/hcp.html.
